# Efficacy of a Web-Enabled, School-Based, Preventative Intervention to Reduce Bullying and Improve Mental Health in Children and Adolescents: Study Protocol for a Cluster Randomized Controlled Trial

**DOI:** 10.3389/fped.2021.628984

**Published:** 2021-04-26

**Authors:** Covadonga M. Díaz-Caneja, Javier Martín-Babarro, Renzo Abregú-Crespo, Miguel Á. Huete-Diego, Marta Giménez-Dasí, Isabel Serrano-Marugán, Celso Arango

**Affiliations:** ^1^Department of Child and Adolescent Psychiatry, Institute of Psychiatry and Mental Health, Hospital General Universitario Gregorio Marañón, Instituto de Investigación Sanitaria Gregorio Marañón (IiSGM), Centro de Investigación Biomédica en Red (CIBER) de Salud Mental (CIBERSAM), School of Medicine, Universidad Complutense, Madrid, Spain; ^2^Department of Research and Psychology in Education, School of Psychology, Universidad Complutense, Madrid, Spain; ^3^School of Psychology, Universidad Nacional de Educación a Distancia, Madrid, Spain; ^4^Consejería de Educación y Juventud, Comunidad de Madrid, Madrid, Spain

**Keywords:** bullying, peer victimization, prevention, school, mental health, cluster randomized controlled trial, special educational needs, disability

## Abstract

**Introduction:** Bullying is a major preventable risk factor for mental disorders. Available evidence suggests school-based interventions reduce bullying prevalence rates. This study aims to test the efficacy of a web-enabled, school-based, multicomponent anti-bullying intervention to prevent school bullying and to assess its effects on mental health and quality of life.

**Methods and analysis:** Cluster randomized controlled trial conducted in 20 publicly funded primary and secondary schools in Madrid, Spain. Schools are randomly allocated to either the intervention arm (*n* = 10) or conventional practices arm (*n* = 10). The web-enabled intervention (LINKlusive) lasts ~12 weeks and consists of three main components: (i) an online training program for teachers and parents, (ii) a web-guided educational program for students, focusing on promoting respect for diversity, empathy, and social skill development, and (iii) a web-guided, teacher-delivered, targeted intervention program for bullying situations identified based on peer-support strategies and individual intervention for those involved (i.e., bullying victims and perpetrators). The primary objective is to compare differences between peer-reported bullying victimization in the intervention and control arms at the end of the intervention. Secondary outcome measures are additional measures of bullying victimization and perpetration, mental health symptoms, self-esteem, and quality of life. A follow-up assessment is conducted 1 year after the end of the intervention. Treatment effects will be tested using multilevel mixed models, adjusting for school-, classroom-, and student-related covariates. Considering the increased bullying rates in children with special educational needs, a specific subgroup analysis will test the efficacy of the intervention on bullying prevalence, mental health, and quality of life in this particularly vulnerable population.

**Ethics and Dissemination:** The Deontology Commission of the School of Psychology, Universidad Complutense in Madrid, Spain reviewed the study protocol and granted ethical approval on 21st January 2019. The results of the trial will be disseminated in relevant peer-reviewed journals and at conferences in the field.

**Trial Registration Number:** ISRCTN15719015.

## Introduction

Bullying is defined as deliberate aggressive behavior inflicted upon a young person by one or more of their peers, repeated over time, and involving a power imbalance favoring the perpetrator(s) (1). There is increasing recognition of bullying as a major public health concern in recent years (2). Approximately 10–25% of children and adolescents have experienced bullying in their lifetime (3, 4). Exposure to bullying in childhood has been associated with a wide range of lifelong adverse health and social outcomes (5–9). For instance, bullying victims show increased risk for anxiety, depressive, and psychotic disorders, poorer physical health, and suicidality both in the short- and long-term, along with poorer educational and vocational outcomes in adulthood (10–15). Several school-based interventions have shown effectiveness in reducing bullying rates by about 20% (16–19). Although individual effect sizes are in the small to moderate range, considering the global prevalence of bullying, the population impact number of these interventions seems compelling (19).

Previous health economic analyses suggest that anti-bullying interventions are also cost-effective, considering the medium- and long-term consequences and the indirect costs (20–22). A recent cost-effectiveness analysis of the Finnish KiVA program reported estimated net savings of more than $3,000 per pupil for a cohort of 200 pupils followed through age 50 (21). However, many of these interventions incur high short-term economic costs and require important time investment by teachers and pupils, thus potentially reducing their applicability in some contexts, especially in low- or medium-income countries. Sustainability of conventional anti-bullying interventions has also been limited so far, with rates of school participation decreasing over time (23). Digitally assisted interventions could help to address some of these limitations and decrease costs by reducing personnel effort and increasing the reach and homogeneity of interventions across settings (24), as previously suggested for other psychosocial or psychological therapies (25–27).

One of the main predictors of being victimized by peers is to be identified as different. Despite intensive efforts to facilitate integration of children with disabilities and special educational or health care needs into mainstream educational systems, they remain at greater risk of both bullying and mental disorders (28–31). In this population, exposure to bullying seems to be a significant mediator between disability and psychosocial stress and mental health (32, 33). For instance, children with autism show a three-fold higher risk of being victimized by peers relative to typically developing individuals (34), and bullying behavior is associated with increased risk of psychological distress, depression, anxiety disorders, and suicidal behavior, as well as poorer educational outcomes in this population (35–41). In fact, one recent longitudinal study found that bullying is a substantial contributor to increased depression rates in young people with autism traits from childhood to adolescence relative to typically developing individuals, even after accounting for genetic risk (42). Some targeted interventions have been developed for children with autism (e.g., peer network interventions, video modeling), and preliminary reports based on very small samples suggest efficacy in reducing peer victimization (43, 44). Some effective parent-assisted social skill training programs for young people with autism, such as the UCLA PEERS Program, also include content that targets bullying, but efficacy on this outcome has not been specifically assessed (45). Despite efforts to achieve a more inclusive educational system (with special needs children mainstreamed into regular schools) and higher bullying rates in these already vulnerable populations, to our knowledge no previous school-based universal prevention program has specifically addressed bullying in youth with special educational needs (SEN) or assessed the efficacy of universal primary preventive anti-bullying interventions in this population.

### Hypotheses and Aims

A web-enabled, school-based, preventive intervention targeting bullying and promoting respect for diversity will be associated with a reduction in bullying prevalence and improved mental health and quality of life in children and adolescents receiving the intervention relative to the control group. Based on evidence from previous studies (19), we hypothesize that these effects will be sustained over follow-up.

A cluster randomized controlled trial tested the intervention relative to a control group, with an expected 1-year follow-up assessment after completion of the intervention (postponed due the COVID-19 pandemic). Twenty public schools enrolling children with SEN in mainstream classrooms were randomly assigned (1:1) to receive the specific anti-bullying intervention or conventional practices. As a secondary goal, we also aimed to test the efficacy of the intervention in the subgroup of children with SEN.

## Methods and Analysis

### Study Design and Setting

**Figure 1:**
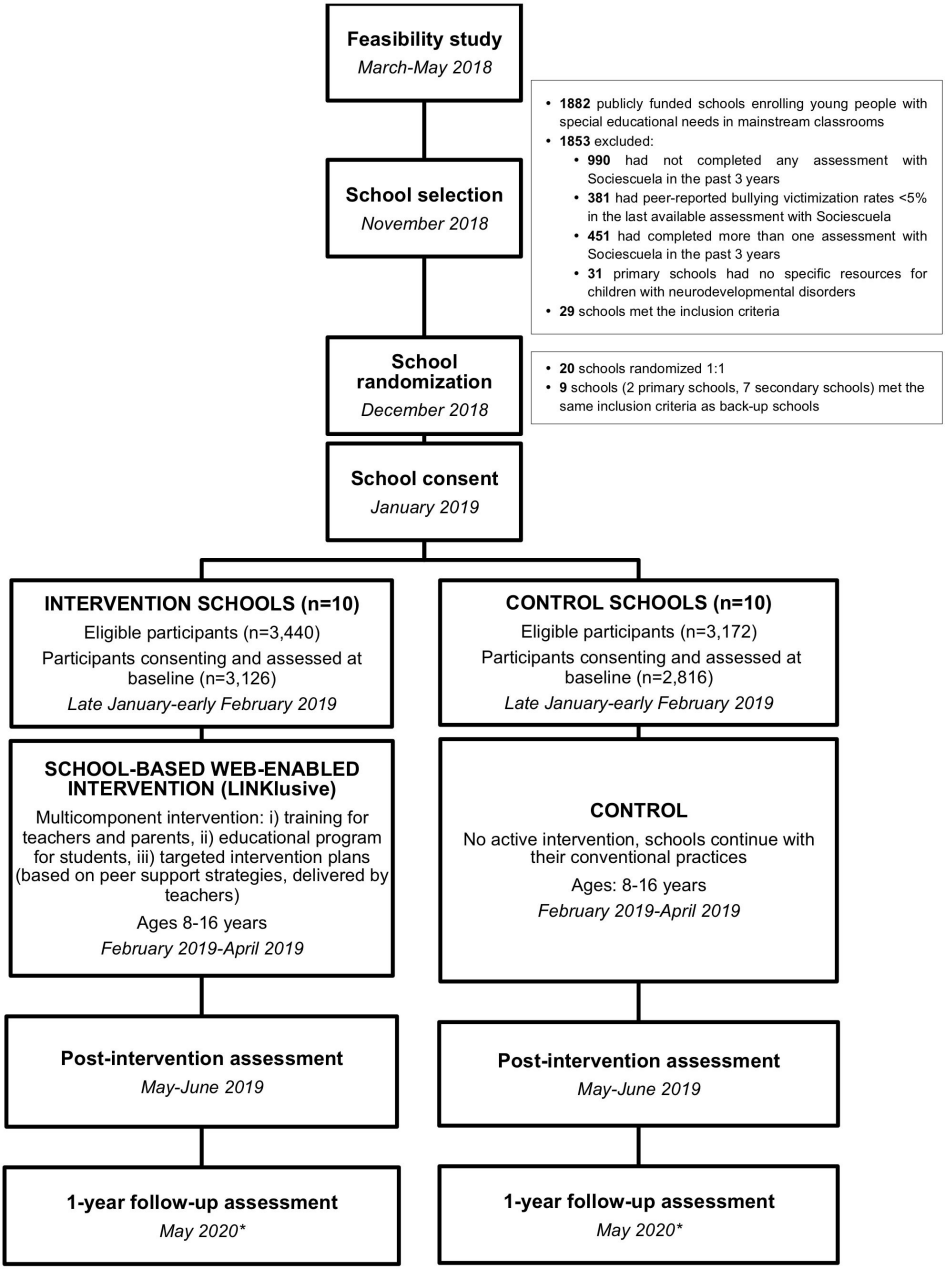
Study design and timelines. *Due to the impact of the COVID-19 pandemic in Spain, the 1-year follow-up assessment has been postponed until the 2020-2021 school year.

This study was a school-based, parallel, cluster randomized controlled trial conducted in publicly funded primary and secondary schools in the Madrid region. The clustering units for the study were schools. See Figure 1 for further details of the study design.

### Study Population and Participant Eligibility

All children and adolescents 8-16 years of age at baseline attending the participating schools could participate in the study. Head teacher consent was required for school participation. Active parental consent was required for use of individual data collected for the study.

### School Recruitment

Figure 1 shows the flowchart for the selection of schools. Schools were selected from among publicly funded primary and secondary schools enrolling youth with SEN in mainstream classrooms in the Madrid region (*n* = 1,882). Inclusion criteria were as follows: (i) at least one previous assessment of bullying with Sociescuela in the past three years, (ii) peer-reported bullying victimization rates of at least 5% in the latest available assessment with Sociescuela, (iii) only one assessment of bullying prevalence using Sociescuela within the past 3 years (in order to have a previous measure of bullying victimization rates, but minimize previous exposure to the tool), and (iv) for primary schools, presence of specific resources for children with neurodevelopmental disorders (i.e., specific classrooms). The latter criterion was used to enrich the sample for children with neurodevelopmental conditions and accounts for the fact that primary schools in the Madrid region are increasingly incorporating so-called “neurodevelopmental disorder classrooms” or “ASD classrooms.” Therefore, students with SEN due to ASD and other neurodevelopmental conditions mainstreamed in regular schools have been preferentially assigned to schools that have such resources in the past few years. These classrooms, which enroll a maximum of 5 students per class, are usually staffed by a teacher specializing in therapeutic pedagogics or speech-language therapy and a social integration technician. Students with SEN enrolled in these classrooms spend between one- and two-thirds of the time with their peer group in their regular classroom and receive complementary pedagogic and social support through specific activities in the specialized support classroom during the remaining school hours. They also receive support to facilitate integration into the regular classroom as well as during unstructured activities such as recess.

This selection process yielded a final sample size of *n* = 29 schools (*n* = 12 primary and *n* = 17 secondary schools).

### Method of Randomization and Allocation Concealment

Multi-stage cluster sampling was done among publicly funded schools in Madrid. In a first stage, we selected all schools fulfilling the abovementioned inclusion criteria. In a second stage, these centers were randomized 1:1 to the intervention and control groups. Ten schools (5 primary schools and 5 secondary schools) were offered participation in each arm of the trial, with a list of back-up schools in case of refusal to participate (2 primary schools and 7 secondary schools). This process would enable participation of about 96 classrooms in each cluster, including about 4,500 students overall (mean number of students per classroom is 23 in primary schools and 25 in secondary schools). Due to the study design, allocation could not be concealed, and the study was not blinded to participating schools or researchers. Psychological and bullying assessments were based on student reports (self-report or peer-report) on an online platform, with students unaware of the study hypotheses and no direct assessments of the outcome measures by members of the research group.

### Intervention

#### Conventional Practices

All students received standard anti-bullying strategies available in the Madrid region, including established school- and region- specific anti-bullying protocols.

**Figure 2:**
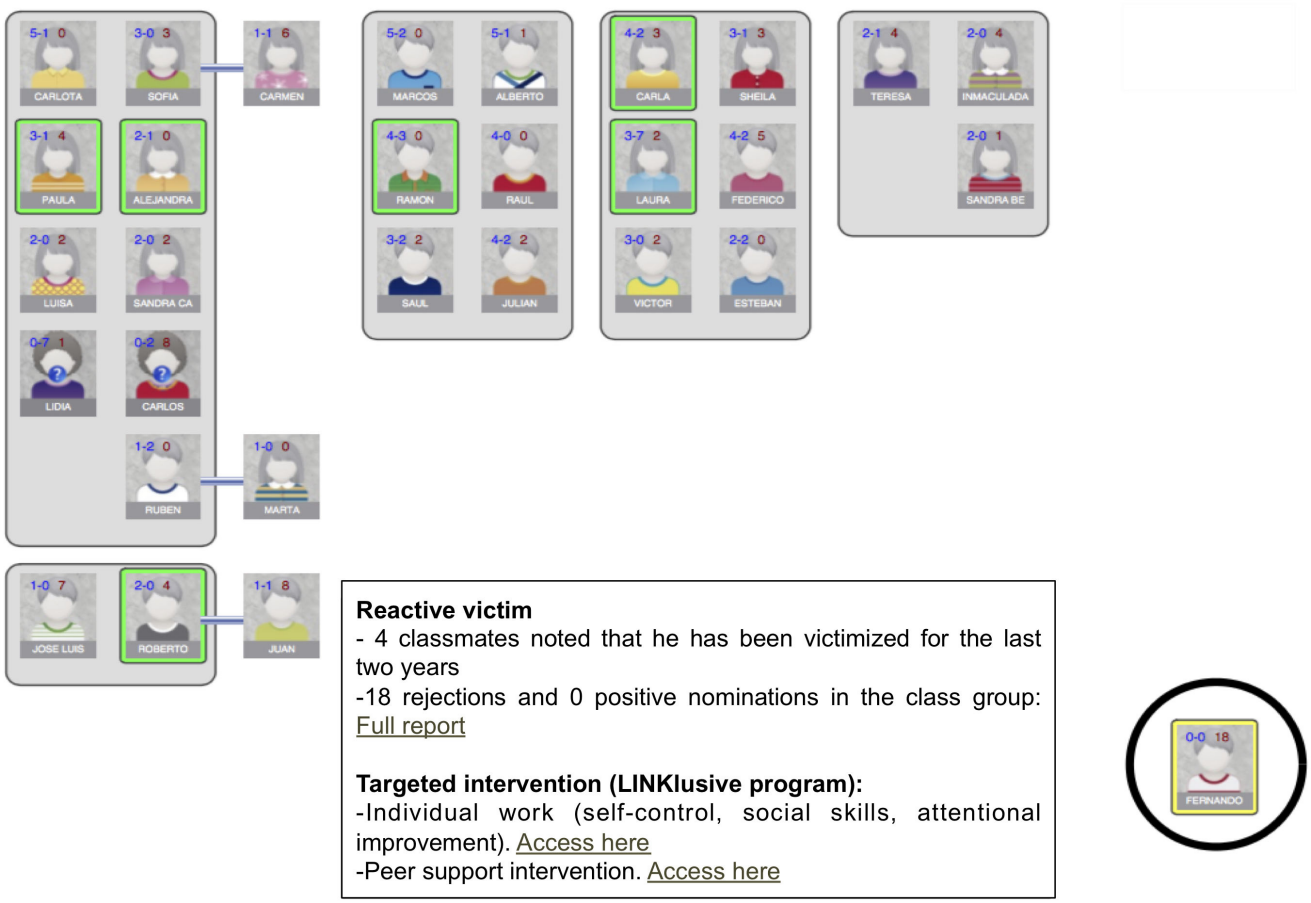
Assessment of peer-reported bullying through Sociescuela. Sociescuela provides teachers with a social map of the classroom, identifying victims, and high-risk cases based on peer reports of victimization, and positive and negative peer nominations (e.g., rejections and positive elections), and bullying perpetrators. In the LINKlusive program, teachers additionally receive links to the individual work program with bullying victims and perpetrators and guidance for the peer-support intervention. Students who might contribute to the peer support intervention can be identified among those who have a positive leadership role in the classroom and do not have a negative attitude toward the victim.

In both groups, an already available online bullying assessment tool (Sociescuela) was used. This online platform enables identification of bullying victims, high-risk cases, and bullying perpetrators by analyzing social networks (social maps or sociograms) within each individual classroom (see Figure 2 for an example) (46). Sociescuela has been widely used in the Madrid region in the past few years for more than 100,000 pupils each year to assess bullying prevalence rates in the region, thus supporting its acceptability (46).

The information provided to teachers by the basic assessment tool (i.e., social maps of the classrooms, identified victims, and the students most suitable for involvement in a peer intervention) may guide interventions using strategies available in the schools, thus potentially reducing differences between the experimental and control groups. Nevertheless, since a Sociescuela assessment had already been conducted at least once at all participating schools before the study and as this assessment method is widely used in the region, for ethical reasons we decided that schools in the control group would also receive these assessment results.

#### Active Intervention: LINKlusive

**Figure 3:**
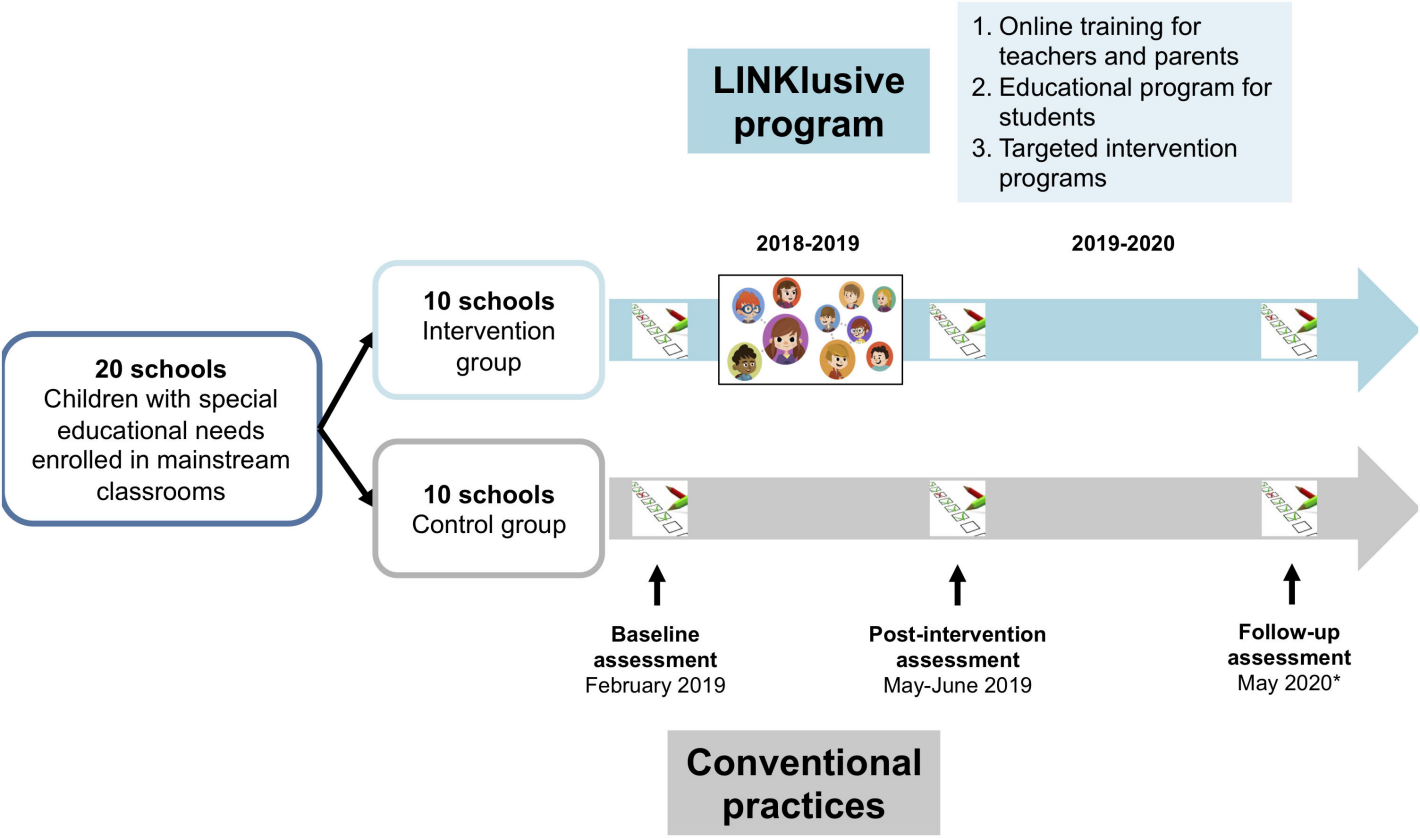
Overview of the LINKlusive intervention program. Due to the impact of the COVID-19 pandemic in Spain, the 1-year follow-up assessment has been postponed until the 2020-2021 school year.

The LINKlusive intervention program (see Figure 3 for further details) builds upon Sociescuela. For the purposes of LINKlusive, the basic Sociescuela online tool was enhanced to facilitate guidance of the targeted interventions and to incorporate the additional educational content and specific materials required for implementing the three components of the LINKlusive program (see below).

The LINKlusive program spans ~12 weeks and consists of three specific components found to be associated with intervention effectiveness in a previous meta-analysis (17):

- Family and teacher training through an online platform. A brief training (about 5 h) was developed for teachers including six teaching units based on practical content with videos, text, and infographics. Each teaching unit has a self-assessment tool to check the level of knowledge acquired about the content. The training program for teachers includes content aimed at increasing knowledge of basic aspects of school climate, how bullying occurs, class social structure, how to establish a peer support intervention, and how to socially energize the group to avoid risk situations and to modify existing ones. It also provides information and guidance on delivering the educational program for students, conducting the assessment of class groups, retrieving and interpreting results, and conducting targeted interventions in identified bullying situations (see subsection iii) below). Researchers also made one visit to experimental centers before study initiation to explain the use of the platform and how to deliver the web-guided educational program for students and targeted interventions.
  A brief training for families was also implemented (about 4 h), consisting of four didactic units aimed at improving their understanding of parental educational styles, facilitating communication and conflict resolution within the family, and providing basic notions about bullying and school climate.
- A universal school-based educational program for students delivered by teachers, with direct web guidance by the LINKlusive platform. This educational program includes content aimed at understanding bullying, promoting respect for diversity, empathy, emotion management, and social skill development (10 sessions lasting about 40 min each during regular school hours), with different activities and materials for primary and secondary students. The work with the didactic units involves teacher-facilitated discussions in small heterogeneous groups of students about the content and videos provided by the online platform. The online platform offers suggestions for teachers on how to establish these working groups (consisting of 4 or 5 students with maximum variability in the following characteristics: sex, sociometric status in the classroom, academic performance, and culture of origin) based on the social network in each classroom.
- A web-guided targeted intervention program for identified bullying situations based on a combination of peer-support strategies and teacher-delivered activities and exercises with bullying victims and perpetrators based on their individual profiles.

In the LINKlusive program, after assessing the social network maps of the classroom provided by Sociescuela and identifying high-risk cases, victims, and potential perpetrators, teachers receive additional instruction and planning for guided personalized interventions based on the social network map in each classroom, by changing classroom organization and using peer-support strategies (see Figure 2) (46, 47).

In the LINKlusive program, teachers also receive specific materials for working with victims and bullying perpetrators, with different content depending on the type of victim (passive or active) and different victim profiles (e.g., children with neurodevelopmental disorders or intellectual disability, LGBT youth).

#### Rationale for the LINKlusive Program

The LINKlusive program combines two universal educational components (aimed at teachers, families, and students) and a targeted intervention component in identified bullying situations. Available evidence shows that comprehensive and systemic approaches that promote the participation of teachers, students, and families may be necessary to address the complex phenomenon of bullying (47).

The two universal educational components of LINKlusive aim at creating a preventive school culture by improving knowledge about bullying in families, teachers, and students and promoting attitudes and strategies to reduce bullying situations and improve school climate. The educational program for teachers includes content to improve their understanding of the bullying scenario and effective intervention tools to act on bullying situations and to improve school climate (including organizational measures for schools and classrooms such as strategies for managing recess and unstructured activities, organizing the classroom and seating arrangements, and promoting teaching styles that may reduce the risk of bullying such as cooperative and project-based learning).

Along the lines of other comprehensive anti-bullying programs (e.g., Friendly Schools Australia, SAVE, OBPP, Kiva) (48–51), the LINKlusive program also incorporates an educational component targeting families, which aims to improve collaboration between the school and families to foster a preventative culture, as well as to provide families with the tools to intervene in bullying situations when their child is involved as a potential victim, bystander, or perpetrator.

The LINKlusive educational program for students fosters a whole class approach based on recognition of the bullying phenomenon and the role of the group in its perpetuation. This is accomplished through activities aimed at raising awareness and promoting favorable and proactive attitudes in classmates, which enable later implementation of specific targeted strategies such as peer support (52). This program also incorporates content to promote respect for diversity, empathy, assertiveness, and other social skills, to prevent bullying by facilitating integration of those perceived as different, who are usually targeted by bullying perpetrators.

The main targeted component of the LINKlusive program focuses on obtaining information on bullying victims and perpetrators and on configuring class groups in a simple and flexible way in order to carry out early targeted interventions based on peer-support strategies and individual work with victims and perpetrators to prevent chronification of bullying situations and incidence of new cases. The strategies to pursue this aim are twofold: (i) modifying the social structure of the group underpinning bullying situations and (ii) improving the social status of the victim.

Several investigations have found that, in most cases, bullying victims and perpetrators—or at least a significant part of the latter—belong to the same class group (53, 54). Victimized students usually experience isolation and have little or no social support in the class group, usually in combination with high peer rejection (55, 56). This association between victimization and social status remains stable if the conditions in the peer group (e.g., social hierarchy, aggressive group norms, and presence of attitudes favoring bullying) are maintained over the academic year. Several strategies based on peer support for the victim have been developed (e.g., peer counseling, befriending, circles of friendship, peer helpers, peer group support), most of which contribute to modifying the social status of the victim in the group to some extent (47, 57, 58). Modifying the group's social architecture and supporting the victim has proven to be effective in reducing bullying (47). It has recently been posited that the effectiveness of these strategies could be increased by a systematic application of network diagnostics to the classroom social structure in order to identify bullying situations and develop interventions (59) such as those used in the LINKlusive program.

In the LINKlusive program, once the Sociescuela bullying assessment has been completed, teachers receive a report including the names of the bullying victims and at-risk students identified in the class and their position within the classroom social map, along with the names of students who could perform best in the peer support intervention (e.g., those who do not reject a vulnerable student in the sociometric evaluation and who, if possible, have a high social status within the group and high levels of prosociality). Teachers also receive an interview script to talk with students potentially involved in the peer-support strategy, which includes guidance on how to request their collaboration to support the victimized student by spending time with him/her between class periods and during recess, as well as making space for that student in his/her group. Finally, classroom areas are organized so as to surround the victim or at-risk student with supportive students.

The teaching staff and the centers are also provided with organizational measures to address the specific social architecture and bullying situations of each particular classroom, with direct guidance on how to form heterogeneous groups, how to modify classroom arrangements, how to create “buffer groups” (especially in centers with more than 4 classes per academic course), how to foster class placements to strengthen peer-support intervention, and how to form class groups for subsequent academic years. The software tool presents a series of screens to help plan and implement these measures.

In the targeted program, strategies to modify the configuration of the social group are complemented with teacher-delivered, web-guided individual work with bullying victims and perpetrators. This work is tailored to the profiles of the victims and the perpetrators and seeks to promote victim resilience after a bullying episode and prevent further episodes by improving social skills, coping mechanisms, and self-esteem, and increasing empathy in those involved in bullying situations. Similar strategies have been implemented and shown to be effective in previous anti-bullying programs (60, 61).

### Feasibility Study

A voluntary feasibility study was conducted during the 2018-2019 school year in six public primary and secondary schools in the Madrid region to test a preliminary version of the LINKlusive web-based intervention program. The intervention was feasible and well accepted by participants. Teachers provided feedback on the materials and the online assessment and intervention platform and collected opinions from students. Teachers suggested several modifications to the report provided by the platform to form the heterogeneous student groups required for delivering the educational program and to the teaching units for secondary education students to suit better their cognitive and social developmental level. After the feasibility study, we implemented some modifications to the report for working with heterogeneous class groups, so that the teacher can request a recombination of the groups if the original proposal is not satisfactory, and we revised the didactic units for secondary education per the teachers' suggestions.

### Study Procedures

Pupils in both groups complete all bullying (i.e., peer- and self-reported measures) and self-reported mental health and quality of life assessments on the web-based platform (www.sociescuela.es) at baseline (January 2019), study endpoint (May-June 2019), and one year after the endpoint (initially expected in May-June 2020) in both treatment groups using tablets (see Figure 1). Due to the impact of the COVID-19 pandemic in Spain, the 1-year follow-up assessment has been postponed until the 2020-2021 school year. The intervention in the experimental group took place between February and May 2019.

Teachers are available in the classroom during assessments for technical questions or clarification. For children with SEN who have comprehension difficulties, class teachers may also supervise data collection, and questionnaires and assessment methods may be adapted as required (e.g., use of visual support strategies for children with autism).

In the experimental group, additional information is collected from families and teachers through the online platform to monitor the use of designated training content. Teachers also report on the degree of completion of the school-based educational program, adherence to intervention recommendations in identified bullying cases, and their satisfaction with the program.

In experimental schools, researchers held two meetings with teaching staff, one before initiation of the intervention to explain the study procedures and present the online platform, and another visit after completion of the intervention to collect feedback. Researchers were also available by phone or e-mail for technical requests or questions regarding delivery of targeted intervention plans in particular situations.

### Measures and Instruments

The main outcome measure of the trial is peer-reported bullying victimization (i.e., at least two peer nominations on the Sociescuela victimization subscale, see below for further details). The study also assesses the following outcome measures: peer-reported bullying perpetration, self-reported victimization and bullying perpetration, mental health symptoms (e.g., internalizing and externalizing symptoms, psychotic-like experiences), self-esteem, and quality of life (see Table 1 for a summary of the variables assessed and the instruments used in the study).

**Table 1:** Variables and instruments used in the LINKlusive study.

**Variable**	**Assessment method**	**Instrument**
Peer-reported bullying victimization and perpetration	Peer-report, social network analysis within the classroom	Sociescuela (46)
Self-reported bullying victimization and perpetration	Self-report, questionnaire	*Ad hoc* questionnaire [adapted from (68)]
General psychopathology, internalizing and externalizing psychopathology	Self-report, questionnaire	Strengths and Difficulties Questionnaire and corresponding subscales (70)
Psychotic-like experiences	Self-report, questionnaire	Community Assessment of Psychic Experiences (CAPE-15) (81, 82)
Self-esteem	Self-report, questionnaire	Rosenberg self-esteem scale (83)
Quality of life	Self-report, questionnaire	KIDSCREEN-10 (86)

*Students completed all assessments through an online platform using tablets. Demographic variables were collected through an ad hoc questionnaire. Teachers also completed questionnaires regarding students with special educational needs, concomitant anti-bullying interventions conducted in schools, and adherence to the LINKlusive intervention components in the intervention group*.

#### Demographic and Social Variables

Student demographic data (age, sex) are collected through the online platform. The socio-economic level of the participating schools will be calculated based on statistical data for the district or town where the school is located published by the regional government of Madrid and the Spanish National Statistics Institute.

#### Students With Special Educational Needs

Additional variables regarding the numbers of pupils with SEN, availability of specific resources for children with neurodevelopmental disorders at each school, and diagnoses for which the pupils receive educational support are collected from the class teachers through the online platform.

#### Bullying Assessment

As recommended by most authors, we included both peer- and self-reported measures of bullying victimization and perpetration in the study. Previous studies have found a low correlation between self- and peer-reported methods in both victims (62–66) and perpetrators (63). Despite the fact that there has been more research using self-reports, some authors suggest that peer-report could enable a more precise identification of bullying situations (63), as it may reduce measurement errors and increase reliability since it is based on multiple informants, while it may also help to overcome the secrecy and code of silence that often surrounds bullying situations (67).

##### Peer-Reported Bullying Victimization and Perpetration: Social Network Analysis

Peer-reported bullying behavior was assessed by analyzing the group structure and social networks within the classroom, where most bullying cases occur (46). This model assumes that bullying is influenced by the social context and not only by the bully-victim interaction. In light of this, Sociescuela is a bullying assessment instrument that uses technique borrowed from social-network analysis for developing social maps (social cognitive mapping, NEGOPY). The rationale and validation data of Sociescuela have been extensively reported elsewhere (46). In brief, students provide peer nominations and ratings of their social preferences, thus providing an accurate picture of the social structure of the class. The instrument is composed of three subscales: (i) a victimization subscale (composed of three items assessing physical, verbal, and relational victimization), (ii) an acceptance subscale (composed of five items based on sociometry assessing positive and negative peer nominations), and (iii) a subscale of perceived attributes (12 items assessing prosociality, withdrawal, and aggressiveness). The subscale of perceived attributes helps identify different victim profiles (e.g., active vs. passive). The instrument also enables identification of bullying perpetrators. The instrument has been validated in a large sample of Spanish children and adolescents and found to have appropriate psychometric properties (46). For purposes of this study, we identify bullying victims and perpetrators as those who receive at least two nominations by their peers as either victims or perpetrators in any of the items of the victimization subscale (62).

Using measures of friendship and frequent interaction, as well as social cognitive mapping, a graphical structure of the group structure is created, which helps identify social groups within the classroom and determine who are not members of any social group, and thus potentially at high risk for peer rejection or victimization (see Figure 2 for an example). This provides additional information on groups of friends in the classroom, peer support for bullies and victims, and the degree of bystander involvement in the bullying situation, thus enabling implementation of targeted or personalized interventions and peer support strategies (46).

##### Self-Reported Bullying Victimization and Perpetration

Peer-reported bullying assessment was complemented by an additional self-reported record of bullying victimization and perpetration experiences using a specifically designed questionnaire composed of 16 items assessing the intensity and frequency of social, verbal, physical, and cyber bullying, and one global item. This questionnaire is based on customary bullying definitions and classifications (1) and previous questionnaires used in other bullying prevention programs (e.g., KiVA) (68) and has previously been used and validated in Spanish students (53).

#### General Psychopathology

General psychopathology is assessed using the Spanish version of the Strengths and Difficulties Questionnaire (SDQ) (69). For purposes of this study, we are using the self-report version of the scale (70). The scale includes five subscales assessing the following dimensions in children and adolescents: emotional symptoms, conduct problems, hyperactivity/inattention, peer relationship problems, and prosocial behavior (i.e., positive social skills). Additionally, scores may also be calculated for two broader subscales assessing internalizing and externalizing symptoms (71). Mean total difficulty scores have shown good predictive validity of clinician-rated mental disorders, with no systematic tendency toward under- or overestimation (72). Depressive symptoms were assessed using nine selected items on the Major Depression Disorder subscale of the Revised Child Anxiety and Depression Scale, which measures child-reported depressive and anxiety symptoms with good validity and reliability (73).

#### Psychotic-Like Symptoms

Psychotic-like experiences are assessed with the Community Assessment of Psychic Experiences (CAPE). The CAPE is a self-report questionnaire that assesses frequency and distress associated with psychotic-like experiences in the general population across three dimensions (positive, negative, and depressive) with good internal consistency and validity (74, 75). It is also considered a valid measure of the extended psychosis phenotype (76). Recently, the positive dimension has been tested as a potential screening tool for young individuals at high-risk for psychosis (77). The CAPE has previously been used in non-clinical samples of adolescents, with appropriate validity (78–80). For purposes of this study, we used the Spanish version of the CAPE-P15 (81), which includes 15 items that assess the positive symptom dimension with good internal consistency (81, 82).

#### Self-Esteem

Self-esteem is assessed with an adapted version of the Rosenberg self-esteem scale (83). The Rosenberg scale is a self-report questionnaire that assesses self-esteem with good internal consistency and test-retest reliability (83). For purposes of this study, we are using a 10-item adapted version of the scale after rephrasing some items on the original scale for use in child and adolescent samples. This version has previously been validated in Spanish children and adolescents and found to have good psychometric properties (84).

#### Quality of Life

Health-related quality of life is assessed with the Spanish version of the KIDSCREEN-10. The KIDSCREEN (with self-report and proxy versions consisting of 52, 27, and 10 items) assesses quality of life according to the young person's mental, physical, and social wellbeing. It was developed and validated for children and adolescents 8-18 years of age in 13 EU countries between 2001 and 2004 (85). The KIDSCREEN-10 is based on a Rasch analysis of the KIDSCREEN-27 version and shows adequate internal consistency, validity, and test-retest reliability (3, 86).

#### Concomitant Intervention and Adherence to Intervention Guidance

The following information is collected through questionnaires specifically designed for the study: additional anti-bullying policies and activities available at all the participating schools, as well as program completion (e.g., proportion of teachers and parents completing the training online, number of classrooms participating in the intervention at each school, number of hours of the educational program completed by each participating classroom) and adherence to intervention recommendations (i.e., adherence to recommendations included in the personalized plans) in the intervention group schools. Students and teachers were asked online how many sessions of the educational program they had completed. Likewise, during the visit to schools after implementation of the intervention program, the researchers collected information on the number of sessions that the teachers had completed in each class group and contrasted this information with that provided by the school management team. As for families, the teaching units had a specific access link for each educational center to record the number of users who accessed each session and the time spent on the training. Based on these data, an adherence score will be calculated for each experimental school.

### Statistical Analyses

#### Sample Size Calculation

Based on an estimated bullying prevalence rate of 3.8% in the largest study conducted in a representative sample of the Spanish adolescent population to date (53), along with data suggesting 20-50% reductions in bullying rates with previous effective interventions (17), and an estimated population sample size of 100,000 students, a sample size of [388 to 2148] students would enable us to detect absolute reductions in prevalence rates of [0.8 to 1.9%] with a power of 80% and a significance level of 95%. In light of potential selection bias, we used a standard mean estimation of the uncertainty coefficient and intragroup correlation, and we duplicated this sample size up to [776 to 4,296] pupils to minimize the risk of a type-I error. To reach an approximate sample size of 4,200 pupils, twenty schools need to be randomized 1:1. This will yield a total of approximately 200 classrooms.

#### Planned Analyses

Demographic, educational, and bullying variables will be compared between the intervention and control groups at baseline using parametric tests, as appropriate. Multi-level mixed model analyses (three levels: school, classroom, and student) will be performed to assess the effect of treatment group (intervention vs. control group) on each outcome variable, while controlling for school socio-economic status, primary vs. secondary school, sex, and age. Additional sensitivity analyses will be performed in the group of students with SEN, where we expect to detect a similar direction of the effects, but with a larger magnitude. In the intervention group, additional analyses will test the potential effect of covariates on the effect size of the intervention, including intensity of the intervention, concomitant activities, primary vs. secondary school, bullying rates at baseline, rural vs. urban environment, and proportion of students with SEN, among others.

### Trial Status

The feasibility study was conducted between March and May 2018. Selection, randomization, and recruitment of schools started in November 2018 and were completed in January 2019. Baseline assessment of the 20 participating schools (10 in each arm) started in late January 2019 and was completed in early February. In the intervention group, the 12-week intervention program was implemented from February through early May 2019. The post-intervention assessment was performed between late May 2019 and early June 2019. The one-year follow-up assessment will take place during the school year 2020-2021.

## Discussion

Bullying is a major preventable risk factor for mental and physical disorders (8, 9, 87). Increasing recognition of the long-term adverse effects of bullying victimization and perpetration has led to the development of several school-based interventions in the past 20 years (18, 88). These interventions have proven effective overall, even when applied for relatively short periods of time (<1 year) (17, 18, 88). Most of these interventions require extensive personnel effort and may incur very high immediate costs. Despite the evidence supporting the cost-effectiveness of these interventions (21), the feasibility and reach of these interventions could be improved by using less time-consuming methods, including web-based interventions. In the past decade, there has been an increase in digitally supported psychosocial interventions, which in some cases show efficacy comparable to face-to-face interventions (e.g., computerized cognitive-behavioral therapy for anxiety and depressive disorders in adolescents and young adults), although there is still insufficient information on the efficacy of some of these interventions and their cost-effectiveness (89). Digitally-enabled interventions could be especially useful in the field of primary prevention and mental health promotion (90), considering that universal preventive strategies need to target a very large number of subjects and should be as ecological as possible (87). Web-enabled interventions also facilitate standardization of interventions across settings and require less economic and time investment, thus facilitating dissemination to wider audiences, including low- or medium-income countries, where human and material resources are usually scarce, while there has been growing access to the Internet and smartphone technology in recent years (90, 91). Furthermore, the fact that children and adolescents are digital natives suggests that digitally delivered and supported interventions may have better acceptability, which is essential for the long-term sustainability of the intervention, beyond an experimental context.

An additional strength of the current intervention is the specific focus on children with special educational needs and other disabilities, including children on the autism spectrum. This study tries to address the lack of studies specifically assessing the efficacy of anti-bullying interventions in these populations, despite clearly higher rates of bullying and other forms of victimization and their negative effects on mental health (28, 34, 42). In this concern, the LINKlusive program adds to previous universal anti-bullying prevention programs by complementing personalized interventions for bullying cases with specific materials for teachers addressing bullying behavior that targets minority groups (e.g., young people with SEN, LGBT youth) and by focusing the student program on the promotion of respect for diversity.

The use of peer-reported measures of bullying behavior and the adaptation of content and assessment methods to youth with SEN are additional advantages of the current study design. Previous studies have shown poor correlations between self- and peer-reported measures of bullying (r ≈ 0.3) (62, 66, 92). The absence of anonymity in self-reports has been indicated as a possible reason for the low correspondence between self-reports and peer nominations (62), and some authors have suggested that some students may be uncomfortable labeling themselves as victims of bullying (93, 94). In children with SEN, especially in those with difficulties interpreting social situations, there may be additional limitations to recognizing and providing an accurate self-report of bullying situations (95), thus further supporting the use of peer-reported measures.

This study has several potential limitations. The most important of these would be the fact that the assessment method used in both treatment groups may also identify victims and guide some kind of intervention in the control group. However, since Sociescuela is widely used in the vast majority of publicly funded schools in the Madrid region, it would seem ethically inappropriate not to provide this information to the control group. The relatively low bullying victimization prevalence rates in our sample at baseline may also preclude detecting a significant effect of the active intervention due to a potential floor effect. Moreover, the need to postpone the follow-up assessment due to the COVID-19 pandemic and the organizational changes derived from social distancing and other preventive measures in schools (including smaller class size and greater supervision of unstructured periods) may affect our ability to detect a sustained effect of the intervention over time. The fact that students may change classrooms or even schools between school years may also influence results of the follow-up assessment. Furthermore, as we conducted our power calculation for the main outcome measure in the whole population, the sample size of the subgroup of students with SEN may be insufficiently powered to detect significant differences, even more so considering the heterogeneity of diagnoses within this group. Therefore, we plan to perform a sensitivity analysis in this subgroup and, based on this proof-of-concept, possibly conduct a larger study specifically testing the efficacy of the intervention in this subgroup in the future. The lack of allocation concealment may have some impact on the results, although, considering that the assessments are collected through the online platform and completed only by the students, we expect this effect to be limited. An additional source of potential bias lies in schools being offered participation after randomization and allocation to the intervention arms, which could have influenced their willingness to participate in the trial. However, this risk was mitigated by the fact that no experimental or control schools refused to participate. The fact that we are testing a multicomponent intervention means we are unable to infer which components may be more efficacious and what may work better for whom. If the LINKlusive program proves to be effective, further studies could assess the specific effect of its main components. Although we have included methods for assessing concomitant interventions in both groups and adherence to the intervention in the intervention group, it is difficult to systematically gather this information, which could affect the results to some extent. Finally, although self-reporting is considered a valid measure of psychopathology, especially in non-clinical contexts, we lack clinician-based measures of diagnosis or psychopathology. However, we do not expect this to significantly affect our main outcome measure. Furthermore, general and dimensional measures of psychopathology may be better suited to assessing short-term changes, and previous evidence suggests good convergent validity with clinician-based measures.

Despite these limitations, this study is the first one to test a web-enabled, user-friendly, anti-bullying intervention with a special focus on promoting diversity and addressing school bullying in mainstreamed young people with special educational needs. As bullying may be considered one of the most prevalent potentially modifiable risk factors for mental disorders (87), the efficacy of such an intervention may provide further support for implementing school-based anti-bullying programs globally, so as to improve life-long health and social outcomes worldwide.

## Ethics Statement

The studies involving human participants were reviewed and approved by the Deontology Commission of the School of Psychology, Universidad Complutense in Madrid, Spain. Written informed consent to participate in this study was provided by the participants' legal guardian/next of kin.

## Author Contributions

CA and CD-C obtained funding for the trial, designed the study, and supervised project implementation. JM-B and MH-D designed the assessment platform, elaborated the educational materials, and developed the personalized intervention plans. MG-D provided support for elaboration of the educational materials. IS-M provided support for implementation of the trial at publicly funded schools in the Madrid region. JM-B and RA-C were in communication with schools to support the assessment and intervention procedures. CD-C wrote a first draft of the manuscript, which was subsequently edited by the rest of the authors. All authors contributed to critical revisions of the paper and have approved the final version of the manuscript for publication.

## Conflict of Interest

CD-C and RA-C have received grant support from Instituto de Salud Carlos III (Spanish Ministry of Science and Innovation). CD-C has also received honoraria from AbbVie, Sanofi and Exeltis. CA has been a consultant to or has received honoraria or grants from Acadia, Angelini, Gedeon Richter, Janssen-Cilag, Lundbeck, Otsuka, Roche, Sage, Servier, Shire, Schering-Plow, Sumitomo Dainippon Pharma, Sunovion, and Takeda. JM-B developed the Sociescuela software, which is managed by a non-profit company. IS-M is employed by Consejería de Educación y Juventud of the Regional Government in Madrid. The remaining authors declare that the research was conducted in the absence of any commercial or financial relationships that could be construed as a potential conflict of interest.
